# Verbascoside-rich *Buddleja davidii* extract in liposomal systems: modulation of release behavior for topical wound treatment

**DOI:** 10.3389/fbioe.2026.1903742

**Published:** 2026-08-26

**Authors:** Adeela Yasmin, Luca Casula, Alessio Pittiu, Claudia Massoni, Francesco Lai, Andrea Carpi, Renzo Dal Monte, Giorgia Barbolini, Paola Brun, Michele Schlich, Chiara Sinico

**Affiliations:** 1 Department of Life and Environmental Sciences, University of Cagliari, Cagliari, Italy; 2 IMEM-BRT Group, Departament d’Enginyeria Química, EEBE, Universitat Politècnica de Catalunya, BarcelonaTech (UPC), Barcelona, Spain; 3 Grup de Caracterització de Materials, Departament de Física, EEBE, Universitat Politècnica de Catalunya, BarcelonaTech (UPC), Barcelona, Spain; 4 Barcelona Research Center in Multiscale Science and Engineering, Universitat Politècnica de Catalunya, BarcelonaTech (UPC), Barcelona, Spain; 5 ABResearch srl, Brendola (VI), Italy; 6 Department of Molecular Medicine, section of Microbiology, University of Padova, Padova, Italy

**Keywords:** antimicrobial, antioxidant, controlled release, liposomes, verbascoside, wound healing

## Abstract

**Introduction:** Chronic wounds represent a major clinical challenge due to their complex pathophysiology, characterized by persistent inflammation, excessive production of reactive oxygen species (ROS), high risk of microbial contamination, and impaired tissue regeneration. Despite several plant extracts offering antioxidant, anti-inflammatory, and antibacterial properties, their therapeutic use is limited by poor stability and rapid clearance of the active compounds from the wound site, particularly in the case of hydrophilic molecules. In this study, a verbascoside-rich extract obtained from *Buddleja davidii* was encapsulated into liposomes for potential wound healing applications.

**Methods:** The formulations were characterized in terms of vesicle size, polydispersity index, and zeta potential using dynamic and electrophoretic light scattering. Encapsulation efficiency and verbascoside release profiles were assessed by HPLC, while antioxidant activity was evaluated by the DPPH assay. Formulation stability was monitored for two months, and in vitro biocompatibility and antioxidant protective effects were investigated in human keratinocytes, while antimicrobial activity was tested against methicillin-resistant Staphylococcus aureus (MRSA) and Escherichia coli.

**Results:** The liposomal formulations modulated the release kinetics of verbascoside, showing a prolonged release of the encapsulated fraction, limiting its rapid dispersion in aqueous environments and enabling sustained availability over time. Encapsulation preserved the antioxidant activity of the extract and ensured excellent in vitro biocompatibility across a wide concentration range. Finally, liposomes showed strong and concentration-dependent antimicrobial activity against MRSA, as well as bacteriostatic responses against E. coli.

**Discussion:** Collectively, these findings highlight liposomal encapsulation as a solid strategy to prolong the release–and thus the residence time–of hydrophilic plant-derived actives while maintaining or potentiating their biological activities in the context of wound healing.

## 1 Introduction

Chronic wounds fail to heal and remain trapped in the inflammatory phase, resulting in the accumulation of pro-inflammatory cytokines and reactive oxygen species (ROS) over extended periods, increasing the risk of bacterial invasion and proliferation (Werdin et al., 2008; Frykberg and Banks, 2015). Due to the multifaceted and complex pathophysiology of chronic wounds, conventional treatments are often ineffective (Han and Ceilley, 2017; Olsson et al., 2019). These issues highlight the need for safer, more accessible, and effective treatments, such as natural, plant-derived therapies that promote healing, gaining attention as safe, viable and cost-effective options for wound management (Shedoeva et al., 2019; Monika et al., 2022). Among these compounds, verbascoside (VRB) is a notable phenylethanoid glycoside found in plants such as *Buddleja davidii* (BD), well known for its strong antibacterial, anti-inflammatory, and antioxidant effects (Vertuani et al., 2011; Marčetić et al., 2025). Despite their therapeutic potential, plant-derived compounds often face limitations related to chemical instability, poor bioavailability, and suboptimal release profiles, requiring advanced drug delivery systems that integrate nanotechnology and biomaterials to address these challenges (Sanjai et al., 2024; Casula et al., 2026a). Phospholipid vesicles are among the most extensively studied nano-formulations for the topical delivery of plant extracts and natural bioactive compounds in the treatment of skin related diseases (De Luca et al., 2024; Jovanović et al., 2025; Maheshwari et al., 2025; Rossi et al., 2025). The biocompatibility of phospholipids and their affinity for biological membranes make them suitable carriers for skin-related applications, with prolonged retention at the application site and controlled release of the encapsulated compounds (Schlich et al., 2016; Wang et al., 2019; Essghaier, 2026).

Previous studies have shown that liposomal encapsulation of VRB efficiently retained more than 90% of the loaded compound for 90 days without degradation (Isacchi et al., 2017), while also enhancing its skin penetration (Sinico et al., 2008) and intracellular uptake (Chronopoulou et al., 2025). In animal pharmacokinetic experiments, both liposomes and chitosan-coated liposomes increased VRB bioavailability by two and fourfold, respectively (Zhou et al., 2018). However, these studies were exclusively focused on the encapsulation of purified VRB, whereas the formulation of complex plant extracts rich in VRB remains largely unexplored despite offering the potential advantages of preserving the natural phytocomplex and the synergistic activity of its bioactive constituents.

In this context, the present work proposes for the first time the encapsulation into liposomes of a VRB-rich *Buddleja davidii* extract, obtained through an innovative biotechnological production process. Considering VRB high hydrophilicity (Crivellari et al., 2018) − which can result in rapid release in aqueous environments such as wound exudate − encapsulation within liposomes offers a strategy to modulate its release rate, while also enhancing its stability, and improving penetration into the skin layers, thereby supporting its therapeutic potential in wound management (Roesken et al., 2000; Hawthorne et al., 2022). Therefore, this approach aims not only to protect the phytocomplex from degradation but also to achieve controlled release while preserving the multiple bioactive components naturally present in the extract. The obtained formulations were characterized in terms of vesicles dimensional properties and zeta potential by DLS and ELS, encapsulation efficiency and release profiles by HPLC, and antioxidant activity through DPPH assay. The stability of the formulations was evaluated for 2 months by monitoring the colloidal properties and VRB content. Finally, biocompatibility and antioxidant activity were evaluated *in vitro* on human keratinocytes (HaCaT), whereas the antibacterial activity was evaluated against methicillin-resistant *Staphylococcus aureus* and *Escherichia coli*. To the best of our knowledge, this is the first study investigating the encapsulation of *Buddleja davidii* extract into liposomes for wound-oriented therapeutic use.

## 2 Materials and methods

### 2.1 Materials

Purified soy phosphatidylcholine (Phospholipon® 90G, P90G) was kindly supplied by Lipoid GmbH (Germany). Verbascoside-rich extract (from plant species *Buddleja davidii; BD*, verbascoside content equal to 59% *w/w*) was generously provided by Active Botanicals Research (ABResearch SRL, Italy) and prepared using an innovative proprietary biotechnological platform: NATRISE (details confidential). The VRB content in the extract was independently validated through HPLC (method described in paragraph 2.4). Pure Verbascoside (>97.0%) from Tokyo Chemical Industry Co., LTD Zwijndrecht, Belgium. Methanol and all the other reagents were of analytical grade and were purchased from Sigma-Aldrich (Milan, Italy).

### 2.2 Preparation of *Buddleja davidii* (BD)-loaded liposomes

Empty and BD-Extract-loaded liposomes were prepared by the direct sonication method (Casula et al., 2026b), according to the composition reported in Table 1. In the first step, BD-Extract (90 mg) was dissolved in 3 mL of 0.2 M citrate buffer at pH 5.0 (CB). After the addition of the lipid components (75 mg P90G and 4.5 or 9 mg cholesterol, CHO, depending on the formulation), the dispersion was sonicated six times (5 s on 3 s off for 5 times) using a titanium probe ultrasonic disintegrator (Soniprep 150 plus, MSE Crowley, UK). The disintegrator tip was submerged directly into the aqueous mixture. As the energy was applied, phospholipids gradually assembled into uniform bilayers, entrapping VRB when it is present. The prepared formulations were stored at 4 °C until further analysis.

**TABLE 1:** Composition and physicochemical characterization (Particle size, polydispersity index (PDI), zeta potential and Encapsulation Efficiency) of empty and BD-Extract loaded liposomes prepared through direct sonication.

​	Composition (mg/mL)			Characterization				
	BD extract	P90G	CHO	Particle size (nm)	PDI	Zeta potential (mV)	Total VRB (mg/mL)	Encapsulation efficiency (%)
Empty-LipoA	-	25	1.5	78 ± 8**	0.30 ± 0.04	−4 ± 0.04	-	-
Empty-LipoB	-	25	3	80 ± 3*	0.34 ± 0.03N	−4 ± 0.54	-	-
BD-Extr-LipoA	30	25	1.5	110 ± 10*	0.27 ± 0.02	−6 ± 0.21	20 ± 1	28 ± 6.8
BD-Extr-LipoB	30	25	3	106 ± 11**	0.36 ± 0.07	−5 ± 0.36	21 ± 1	19 ± 3.4

BD, *buddleja davidii*; P90G, soy phosphatidylcholine (Phospholipon® 90G); CHO, cholesterol.

Results are stated as mean ± standard deviation (n = 3). Asterisks indicate statistically significant differences between the corresponding empty and loaded formulation (*p < 0.05, **p < 0.01).

### 2.3 Liposomes’ characterization

The obtained liposomal formulations were characterized by dynamic and electrophoretic light scattering (DLS; ELS) using a Zetasizer nano (Malvern Panalytical, Worcestershire, UK). The average diameter, polydispersity index (PDI) and zeta potential of both empty and BD-Extract loaded liposomes were measured in triplicate at 25 °C ± 0.1 °C after dilution (1:100 *v/v*) with milli-Q water. The stability of the samples was monitored for 60 days by measuring the mentioned colloidal parameters via DLS and ELS, and VRB concentration via HPLC.

Exhaustive dialysis was used to remove unencapsulated VRB from the formulation, in order to estimate the VRB encapsulation efficiency (EE%) of BD-Extract loaded liposomes (Casula et al., 2024). For this purpose, 2 mL of BD-Extract loaded liposomes were loaded into dialysis tubes (molecular weight cut-off (MWCO) 300 kDa; Spectrum Laboratories Inc., Breda, The Netherlands) and dialysed for 2 h against 400 mL of CB under stirring to promote the diffusion of un-entrapped verbascoside into the buffer. Both purified and unpurified samples were diluted with methanol to disrupt the vesicles and analyzed using HPLC. The encapsulation efficiency (EE%) of verbascoside was determined as follows:

$$EE\%=\frac{VRB\;in\;purified\;samples}{VRB\;in\;unpurified\;samples}x\;100\%$$

### 2.4 Quantitative analysis

VRB quantitative analysis was carried using a previously developed method with slight modifications (Sinico et al., 2008). Samples were analysed using an Alliance 2690 (Waters Corp, Milford, MA, USA) high-pressure liquid chromatograph (HPLC), equipped with a photodiode array detector and a computer controlling system with Empower 3. VRB was quantified at λ = 329 nm using a Cortecs C18 column (2.7 µm, 4.6 × 100 mm, Waters) and an isocratic elution with mobile phase of acetonitrile:water:acetic acid (74.5:25.35:0.15 v/v) run at 0.5 mL/min at 30 °C, RT = 2.15 min. Pure VRB standard solutions were used to generate a standard calibration curve (R^2^ = 0.999). VRB limit of quantification (LOQ) was 20 ng, whereas the limit of detection (LOD) was 1 ng.

### 2.5 Antioxidant activity

The antioxidant ability of the samples was measured by using the 2,2-diphenyl-1-picrylhydrazyl (DPPH) method (Dessì et al., 2026). Purposely, 2 mg of DPPH was dissolved in 50 mL of methanol and stored in the dark. 10 μL of each sample (BD-extract solution in CB (BD-Extr-sol), empty and BD-extract-loaded liposomes) were treated with 990 µL of DPPH solution, briefly vortexed and then incubated in the dark for 30 min. After incubation, the absorbance was read at 517 nm using a Synergy 4 multiplate reader (BioTek, Winooski, USA). The antioxidant activity was calculated using the following formula:

$$Antioxidant\;activity\;\left(\%\right)=\frac{\left(CTRL\;absorbance-SAMPLE\;absorbance\right)}{CTRL\;absorbance}x\;100\%$$

where CTRL absorbance (control) represented the absorbance of CB treated with DPPH.

### 2.6 *In-vitro* release studies

*In vitro* release studies were carried out using a dialysis method to investigate VRB release profile in phosphate-buffered saline (PBS, pH 7.4) at 37 °C under constant stirring (Kurt and Aslan, 2025). Briefly, 1.5 mL of purified or unpurified BD-extract–loaded liposomes, as well as BD-Extr-sol, were loaded into dialysis tubes (MWCO 300 kDa) and immersed in 100 mL of PBS to maintain sink conditions. At predetermined time points (30 min, 1 h, 2 h, 4 h, 6 h and 8 h), aliquots (1 mL) were withdrawn from the release medium and replaced with an equal volume of fresh PBS to maintain sink conditions. The amount of released VRB was quantified by HPLC. Release profiles were fitted into the following mathematical release models (Zero-order, First-order, Pseudo-second-order, Higuchi, Hixson–Crowell, Korsmeyer–Peppas, and Weibull) and R^2^ and kinetic parameters were calculated using Microsoft Excel (Sontakke et al., 2022).

### 2.7 Cellular studies

#### 2.7.1 Biocompatibility

The biocompatibility of the obtained formulations was assessed on human cultured keratinocyte cells (HaCAT), obtained by ATCC. The experiment was carried out in accordance with the standard MTT assay protocol. Culture medium was composed of Dulbecco Modified Eagle Medium (DMEM, high glucose, Sigma Aldrich, Milan, Italy) with the addition of 2 mM L-glutamine, 10% fetal bovine serum (FBS), 100 units/mL penicillin and 0.1 mg/mL of streptomycin. Cells were incubated at 37 °C in a 5% CO_2_ atmosphere. Cells were seeded in 96-well plates (1 × 10^4^ cells/well) 24 h before the experiment. The following day, the seeded cells were treated with BD-Extr-sol or BD-Extr-LipoA diluted in complete medium. The extract concentrations tested were between 1 and 250 μg/mL (equivalent to VRB concentrations between 0.59 and 147 μg/mL). Cells were kept under treatment for 4 or 24 h in normal growth conditions. Subsequently, treatment was replaced with a 0.25 mg/mL solution of MTT reagent (3-(4,5-dimethyl-2-thiazolyl)-2,5-diphenyl-2H-tetrazolium bromide, Sigma Aldrich) in PBS and, after 3 h, formazan crystals were dissolved in ethanol. Absorbance at 570 nm was measured using an Infinity 200 plate reader (Tecan Trading AG, Switzerland). Data is reported as the percentage of viable cells considering untreated cells as 100% (average ± standard deviation, n = 5).

#### 2.7.2 Antioxidant effect assessment *in vitro*

BD-Extr-sol and BD-Extr-LipoA antioxidant effect were assessed on HaCAT cells using the reactive oxygen species (ROS) probe 2′,7′-Dichlorodihydrofluorescein diacetate (DCFH-DA) (Ilgaz et al., 2025). Briefly, 2 ×10^4^ cells/well were seeded in 96-well plates and incubated for 48 h. After 48 h, cells were treated with free extract or with extract-loaded liposomes at different extract concentrations (100 and 250 μg/mL, corresponding to approximately 59 and 147.5 μg/mL VRB) doses for 4 h. Subsequently, treatment was first replaced with DCFDA in PBS and, after 30 min, with hydrogen peroxide in PBS for 120 min. Once all incubation steps have been completed, intracellular oxidation of the probe due to ROS was determined by fluorescence (480–530 nm) using BioTek Synergy LX (Agilent Technologies, Santa Clara, USA). The following equation was used to calculate the intracellular ROS percentage:

$$ROS\;\%=\frac{\left(\text{Ft}-\text{Fblank}\right)}{\left(\text{Fut}-\text{Fblank}\right)\;}x\;100\%$$

where Ft: fluorescence of liposomes/extract treated well; Fblank: background fluorescence of cells not exposed to DCFDA; Fut: fluorescence of cells untreated with liposomes/extract or hydrogen peroxide. Data is reported as the average ± standard deviation (n = 5).

### 2.8 Antibacterial studies

#### 2.8.1 Bacterial strains and culture conditions

To assess the antimicrobial activities, two bacterial strains were used: methicillin-resistant *Staphylococcus aureus* (MRSA; ATCC 33592), a Gram-positive bacterium, and *Escherichia coli* (*E. coli*; ATCC 25922), a Gram-negative bacterium. For both bacterial species, the inoculum was prepared by growing a single colony in nutrient-rich microbial growth broth (BD, Milan, Italy) at 37 °C with shaking at 150 rpm for 16 h. Subsequently, the optical density of the cultures was measured, and dilutions were prepared in Mueller-Hinton broth (MHB, BD) to obtain concentrations suitable for the experiments.

#### 2.8.2 Resazurin reduction assay

The resazurin reduction assay was used to determine bacterial cell viability and to calculate the minimum inhibitory concentration (MIC). Briefly, freshly prepared bacterial cultures were seeded at 10^4^ CFU/mL in 100 μL/well MHB into Nunc™ 96-well optical-bottom black microplates (ThermoFisher, Milan, Italy). Based on previous experiments conducted in our laboratory (data not shown), the ranges of verbascoside (VRB) concentrations showing antimicrobial activity against both MRSA and *E. coli* were selected. Specifically, in this study, MRSA cultures were incubated with VRB concentrations ranging from 0 to 1.44 mg/mL; *E. coli* cultures were incubated with VRB concentrations ranging from 0 to 2.50 mg/mL. Purified and unpurified BD-extract–loaded liposomes (see Section 2.3) were tested (BD-Extr-LipoA-P and BD-Extr-LipoA-NP, respectively), as well as a solution of the BD extract in citrate buffer (BD-Extr-sol). Positive controls, namely, vancomycin (VAN) and ciprofloxacin (CIP) were included in the experiments.

Since the purified and unpurified formulations contained different VRB concentrations, the volumes of liposomes used in the antimicrobial assays were adjusted accordingly to ensure equivalent VRB levels in the bacterial cultures. Bacterial cultures incubated with empty liposomes (Empty-LipoA) or vehicle (CB at the highest volume used) served as controls for liposomal formulations and BD-Extr-sol, respectively. Non-treated (NT) bacteria served as negative controls.

The plates were incubated at 37 °C for 16 h. Subsequently, resazurin (Merck, Milan, Italy) was added at a final concentration of 0.05 μg/mL, and the plates were incubated for 3 h at 37 °C. Fluorescence signals (Ex 570 nm, Em 590 nm) were measured using a microplate reader (ThermoScientific, Milan, Italy). The signals were plotted as percentages of the reduced resazurin relative to the values recorded in NT samples.

#### 2.8.3 Kinetic bacterial growth curve

To determine whether the liposomal formulations reported bacteriostatic or bactericidal activities, bacterial growth was monitored over time. As previously reported, Empty-LipoA and CB served as controls and were used at the same volumes as the liposomes or extract solution, respectively. Freshly prepared bacterial cultures (10^4^ CFU/mL) were transferred to 96-well plates and added with the tested samples. Plates were incubated at 37 °C for 24 h in a microplate reader (ThermoScientific). Optical density (OD_600_) was recorded every 30 min after 5 s of gentle shaking at 150 rpm. For each point, the average blank value (MHB only) was subtracted from all other measurements before generating the growth curves.

#### 2.8.4 Colony count assay

The colony count assay was used to confirm the bactericidal activity of the liposomal formulations. Bacteria were cultured at 37 °C for 16 h with or without the tested samples and the vehicle. At the end of incubation, bacterial cultures were properly diluted in BHI medium, uniformly spread on LB agar plates, and incubated at 37 °C for 16 h. At the end of incubation, CFUs were enumerated.

### 2.9 Statistical analysis

Student’s t-test was used to assess the significant differences between the two groups. One-way ANOVA with *post hoc* Tukey HSD test was used to evaluate statistical differences between groups comparing multiple means. Two-way ANOVA followed by Bonferroni multiple-comparison *post hoc* test was employed to evaluate differences in antimicrobial analyses. p values < 0.05 were considered statistically significant.

## 3 Results and discussion

### 3.1 Liposomes’ preparation and characterisation

The effective topical use of plant-derived hydrophilic bioactive compounds is often limited by poor stability and retention at the application site (Rahman et al., 2020). To overcome this issue, liposomal formulations encapsulating a *Buddleja davidii* extract were prepared with different phospholipid:cholesterol ratios (Table 1). P90G is a purified (>90%) commercial mixture of soybean phosphatidylcholines, mainly composed of unsaturated fatty acids characterized by low transition temperatures (Pittiu et al., 2024). As such, their assembly into vesicles is favored, as well as their interactions with lipids of the stratum corneum (Coderch et al., 2000). On the other hand, excessive membrane fluidity could result in unwanted high permeability to hydrophilic molecules, such as VRB. Therefore, cholesterol was added to the lipid mixture at two different concentrations to stabilize the bilayers, reducing the excessive permeability to achieve higher encapsulation efficiencies and controlled release (Nsairat et al., 2022). Considering the well-known VRB degradation at pH values ≥ 7 (D’Imperio et al., 2014; Wisuitiprot et al., 2022), CB at pH 5 was used to prepare the formulations.

BD-Extract-loaded vesicles showed larger particle sizes, 110 ± 13 nm and 106 ± 14 nm, respectively, for BD-Extr-LipoA and BD-Extr-LipoB than their corresponding empty formulations, as 78 ± 9 nm (Empty-LipoA) and 80 ± 3 nm for (Empty-LipoB), with slightly higher–but not significant–polydispersity index for the samples with the higher cholesterol amount. Nonetheless, all formulations exhibited PDI values of approximately 0.3, indicating acceptable size homogeneity. This is considered a suitable value for lipid-based drug delivery systems, despite the lack of specific regulatory guidelines defining acceptable PDI ranges for different applications (Danaei et al., 2018).

Moreover, all the samples had moderately negative zeta potential values, which ranged from −4 to −6 mV. These values are in agreement with those obtained by Chronopoulou et al., who prepared phosphatidylcholine liposomes loaded with VRB by microfluidics, using a capillary hydrodynamic flow-focusing device (Chronopoulou et al., 2025).

Purification through exhaustive dialysis, followed by HPLC quantification, revealed slightly higher encapsulation efficiency values for BD-Extr-LipoA compared to BD-Extr-LipoB (28% ± 7% vs. 19% ± 3%; Table 1). Considering the high aqueous solubility of the hydrophilic compounds, VRB is expected to dissolve in the external aqueous phase during liposome preparation, while a smaller amount is incorporated in the vesicles aqueous core (Xu et al., 2012). Isacchi et al. reported comparable entrapment efficiencies (approximately 30%) for pure verbascoside loaded in liposomes prepared through the thin film hydration method (Isacchi et al., 2017). It is important to note that the direct sonication method employed in our work offers several advantages over the conventional thin-film hydration method, including a simpler, one-step preparation process, higher reproducibility, and greater potential for scale-up. Moreover, in the context of wound treatment, relatively low encapsulation efficiencies might not be considered a limitation, as the non-encapsulated actives is immediately available to interact with the partially or completely compromised stratum corneum.

**FIGURE 1:**
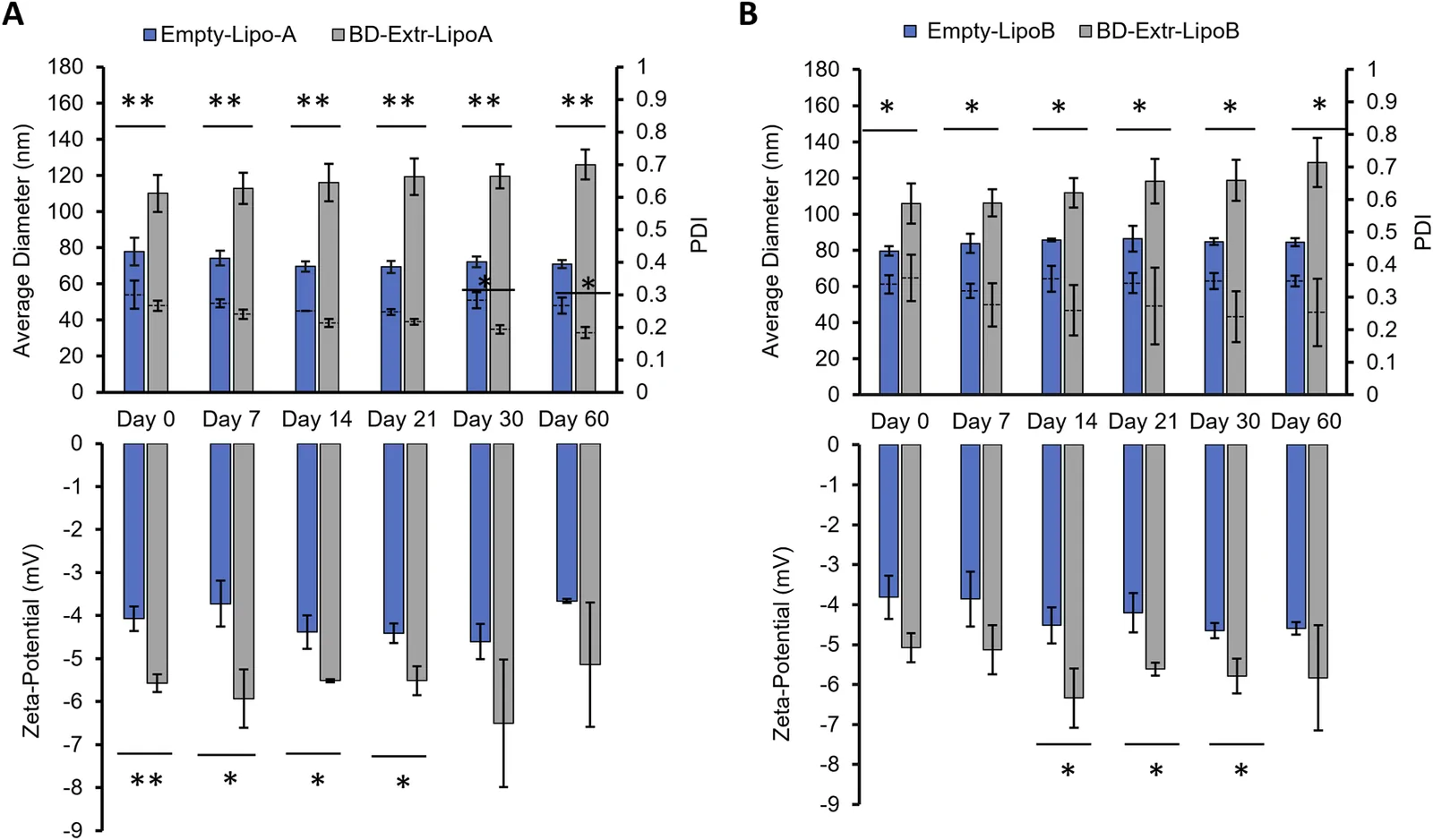
Colloidal stability of liposomal formulations stored at 4 °C for 60 days in terms of average size (nm), Polydispersity Index (PDI) and zeta potential (mV), for **(A)** Empty and BD-Extr-loaded Liposomes A **(B)** Empty and BD-Extr-loaded Liposomes B. Results are stated as mean ± standard deviation (n = 3). Asterisks indicate statistically significant differences between the corresponding empty and loaded formulation at the same time-point (*p < 0.05, **p < 0.01, One-way ANOVA with Tukey’s *post hoc* test).

The colloidal parameters and VRB concentration were monitored over 60 days to evaluate the stability of the obtained formulations stored at 4 °C. As shown in Figure 1, the average particle size of empty liposomes remained essentially unchanged throughout the study, whereas the BD-Extract-loaded liposomes, particularly BD-Extr-LipoB, displayed a slight increase in size that was not statistically significant. PDI values remained low and largely unchanged, indicating homogeneous vesicle populations and minimal aggregation. Although the absolute values of the zeta potential were moderately negative, ranging approximately between −5 and −6 mV, they were sufficient to maintain colloidal stability over time.

Chemical stability was also assessed by monitoring VRB concentration in the liposomal formulations (BD-Extr-LipoA, BD-Extr-LipoB) and in the corresponding BD-Extr-sol, used as control. Literature data reports several examples of increasing VRB stability and reducing degradation kinetics under storage conditions by encapsulation in lipid-based nanocarriers (e.g., liposomes, lipid nanocapsules) (Sinico et al., 2008; Isacchi et al., 2017; Wu et al., 2023).

In our study, VRB concentration remained constant over the entire monitoring period in both liposomal formulations and the BD-Extr-sol, with no statistically significant degradation detected by HPLC analysis (Supplementary Figure S1). These findings are partially in contrast with Cardullo et al., who reported a retainment of only 58.7% of initial VRB content in a *Lantana camara* extract solution after 2 months at 5 °C, whereas an analogous phytosome formulation retained 96.9% of VRB over the same period (Cardullo et al., 2025). These differences in the stability of the extract solutions may be attributable to variations in extract composition and the presence of co-occurring phytochemicals with intrinsic stabilizing effects (Lachowicz-Wiśniewska et al., 2025). In particular, bioactive compounds in crude extracts can engage in hydrogen bonding, and other non-covalent interactions that can attenuate oxidative and hydrolytic degradation, thus providing a degree of mutual protection (Nemli et al., 2024). Taken together, these findings suggest that liposomal encapsulation *per se* successfully preserves VRB integrity, and that extract matrix components may further modulate stability, underscoring the importance of both delivery system design and source extract composition in the development of stable nanomedicine formulations for hydrophilic phytochemicals.

### 3.2 Antioxidant properties of BD-extract and liposomes

Oxidative stress is recognized as a critical factor influencing wound healing, as excessive production of reactive oxygen species (ROS) can induce cellular damage and impair tissue repair. Consequently, strategies aimed at reducing ROS levels via antioxidant systems may mitigate oxidative stress–mediated damage and promote more efficient wound healing (Schäfer and Werner, 2008; Deng et al., 2021).

**FIGURE 2:**
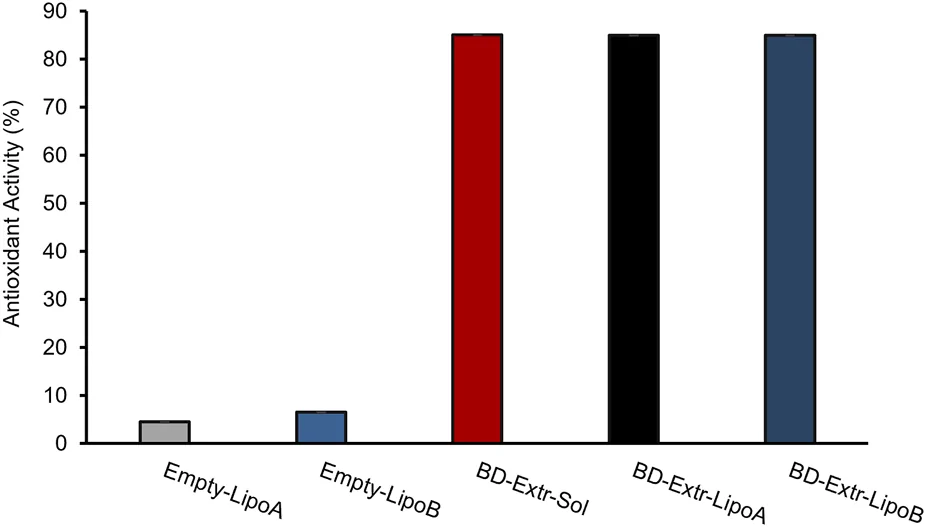
Antioxidant activities (%) of BD-Extract and liposomal formulations, measured using DPPH assay. The tested groups include BD-Extract Solution (BD-Extr-Sol), Empty-LipoA, Empty-LipoB, BD-Extr-Lipo A, and BD-Extr-LipoB. Results are stated as mean ± standard deviation (n = 3).

In the current study, the antioxidant activity of the samples was assessed through the DPPH assay (Figure 2). The DPPH assay determines the antioxidant capacity of a compound by measuring its ability to reduce the stable nitrogen-centered radical DPPH. Upon reaction, the radical is neutralized through electron or hydrogen donation, causing a colour change and a corresponding decrease in absorbance, which reflects the compound’s free radical scavenging activity (Gulcin and Alwasel, 2023).

Both the BD-Extr-sol and its liposomal formulations (BD-Extr-LipoA and BD-Extr-LipoB) demonstrated strong antioxidant activity, exceeding 85%, indicating that verbascoside as well as other bioactive compounds present in the extract retain their radical-scavenging capacity after encapsulation (no statistical difference) (Ahmad et al., 2009; Chen et al., 2025). Empty liposomes showed minimal activity (5%–7%), attributable to the phospholipid components, confirming that the carrier itself does not significantly contribute to antioxidant effects. These findings indicate that liposomal encapsulation effectively preserves the antioxidant activity of the extract, with the fabrication process (i.e., sonication) having no detrimental effect on the functional integrity of the bioactive compounds (Casula et al., 2025).

The free radical scavenging activity of verbascoside is closely related to the presence of free hydroxyl groups within its glucose moiety, which are recognized as key structural features contributing to its antioxidant behavior (Reid et al., 2019; Wu et al., 2023; Rossi et al., 2024).

### 3.3 *In-vitro* release studies

Sustained release is a particularly favorable prerequisite for wound healing systems as it enhances residence time of bioactive compounds in the wound environment (Kurt and Aslan, 2025; Tu et al., 2025).

**FIGURE 3:**
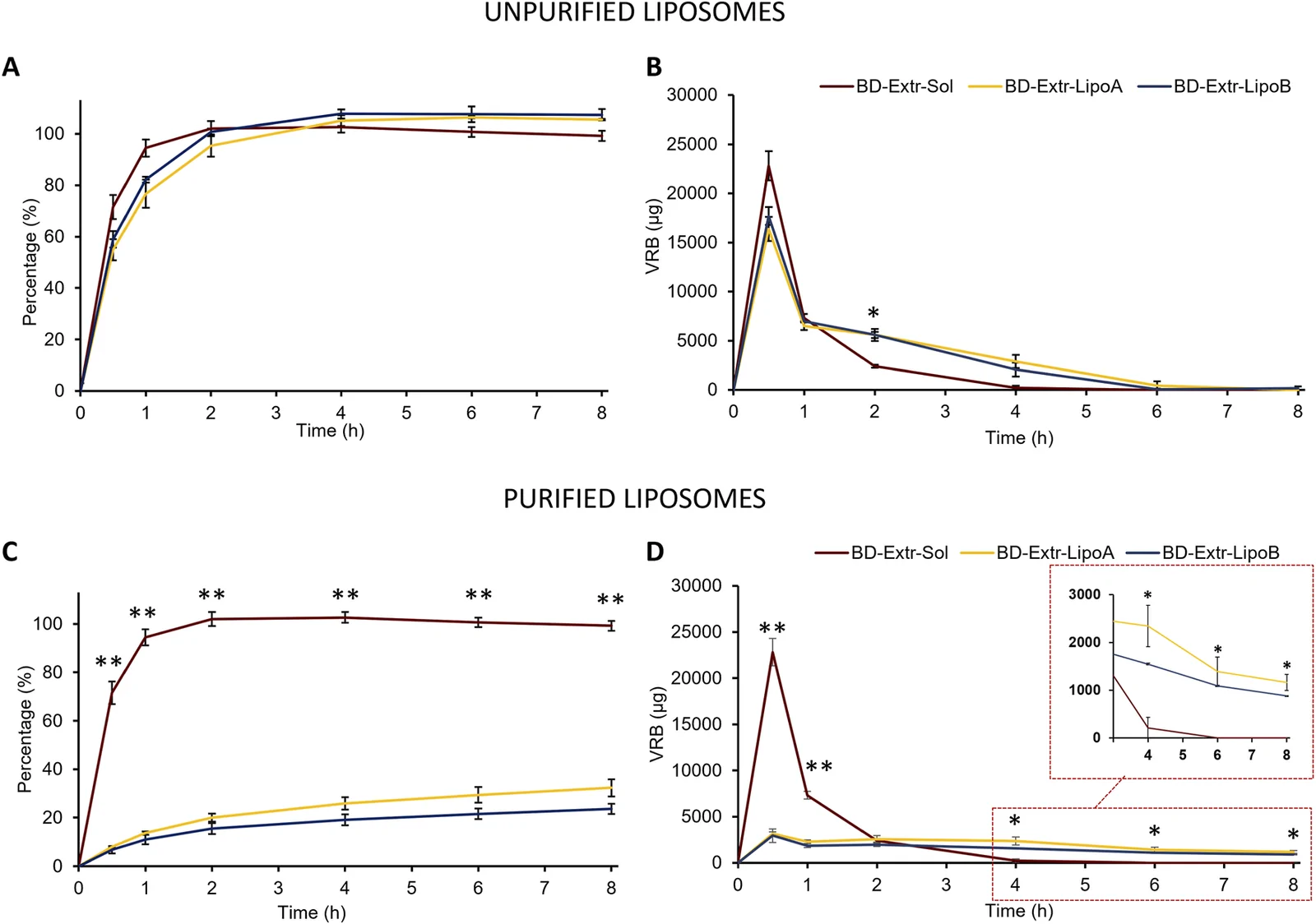
*In vitro* release profile of verbascoside (VRB) from BD-Extract solutions (BD-Extr-sol), BD-Extr-LipoA, and BD-Extr-LipoB, both as unpurified (on the top) and purified (on the bottom) over 8 h in PBS at 37 °C. Release curves are presented as percentage cumulative release (left side, **(A,C)**) and individual release (µg VRB at each time point; right side, **(B,D)**). Data are expressed as mean ± standard deviation (n = 3). Asterisks indicate statistically significant differences between BD-Extr-Sol vs. BD-Extr-loaded Liposomes (A or B) in terms of VRB release (%) or release amount (µg) at the same time-point (*p < 0.05; **p < 0.01, One-way ANOVA with Tukey’s *post hoc* test).

The *in vitro* release profile of VRB was therefore evaluated at 37 °C in PBS (pH 7.4) for the BD-Extr-sol and for BD-Extract-loaded liposomal formulations. Release studies were performed using both unpurified liposomal dispersions (which contain free and encapsulated VRB), and purified liposomes, in which non-encapsulated VRB was removed prior to analysis through exhaustive dialysis as detailed in paragraph 2.3. This experimental approach enabled discrimination between the fraction of VRB immediately available in solution (*initial dose, ID*) and the fraction that is slowly released from the vesicles (*maintenance dose, MD*), allowing a clearer assessment of the contribution of liposomal encapsulation to the overall release behavior and sustained delivery of VRB (Pittiu et al., 2025). The release curves are presented in Figure 3 as percentage of cumulative release (left side, A and C) and as the amount of VRB released at each time point (right side, B and D).

VRB release from unpurified liposomes and the BD-Extr-sol showed a quick and almost complete release in the first 2 hours of testing (Figure 3A), with a pronounced burst effect at 30 min, which can be attributed to the instantaneous diffusion from the membrane of VRB molecules that are freely available (Figure 3B). Particularly, although not statistically different, the BD-Extr-sol shows a relatively higher amount of released VRB at 30 min compared to the liposomal formulations, and a fast decline to zero in the following hours of testing. By contrast, the liposomal formulations displayed a less pronounced burst effect and a more gradual decrease in VRB concentration over time, indicating a delayed release of the compound relative to the BD-Extr-sol. On this basis, purified liposomes were used to specifically evaluate the release behavior of the vesicle-associated VRB fraction, minimizing the contribution of the free unencapsulated molecules.

To further elucidate the release mechanisms governing the different formulations, the experimental data were fitted to seven widely accepted kinetic models, namely, Zero-order, First-order, Pseudo-second-order, Higuchi, Hixson-Crowell, Korsmeyer-Peppas and Weibull models (Supplementary Table S1). The mathematical analysis revealed two clearly distinct release mechanisms directly associated with the purification status of the formulations and, consequently, with their encapsulation efficiency.

The free extract solution (BD-Extr-sol) and both unpurified liposomal formulations exhibited the highest correlation with the Weibull model (R^2^ ≥ 0.991), followed by the First-order model (R^2^ ≥ 0.979). The Weibull shape parameter (β) ranged from 0.26 to 0.32 (β < 0.75), mathematically describing an asymmetric release profile characterized by a pronounced initial burst release. This behaviour is consistent with the moderate encapsulation efficiencies measured for the liposomes (28% for BD-Extr-LipoA and 19% for BD-Extr-LipoB), indicating that approximately 72%–81% of the hydrophilic verbascoside remained freely dissolved in the external aqueous phase. Consequently, the release of these formulations is predominantly concentration-dependent and governed by unrestricted diffusion across the dialysis membrane, while the contribution of vesicle-mediated transport remains largely masked by the excess of freely available compound. As expected, a markedly different kinetic behaviour emerged after removal of the non-encapsulated fraction. In purified BD-Extr-LipoA and BD-Extr-LipoB, both the First-order and Weibull models no longer adequately described the experimental data, whereas the Higuchi (R^2^ ≥ 0.994) and Korsmeyer-Peppas (R^2^ ≥ 0.997) models provided an almost perfect fit. Moreover, the diffusional exponent (n) obtained from the Korsmeyer-Peppas model was 0.49 for BD-Extr-LipoA and 0.51 for BD-Extr-LipoB. For spherical lipid vesicles, values of n ≈ 0.5 are characteristic of Fickian diffusion, providing strong mathematical evidence that verbascoside is progressively released by diffusion through an intact phospholipid bilayer rather than by concentration-driven leakage (Schifone et al., 2026). The kinetic modelling validates the experimental release profiles (Figure 3C), as both formulations exhibited only a minimal burst effect at 30 min, followed by a sustained and nearly constant release phase over several hours, maintaining detectable concentrations in the receiving medium up to 8 h (Figure 3D), reflecting the predominance of the encapsulated VRB that is gradually released from the liposomal system over time.

Furthermore, the direct sonication method produced homogeneous vesicles, with mean diameters of approximately 106–110 nm and low polydispersity indexes (PDI ≈ 0.27–0.36). Such narrow size distributions provide a uniform surface-area-to-volume ratio throughout the formulation, contributing to the reproducible Fickian diffusion profile observed for the purified liposomal systems throughout the 8-h release experiment.

These findings highlight the ability of the developed formulation to provide a dual release behavior (Eloy et al., 2014; Alomrani et al., 2019), in which an initially free fraction of VRB ensures an early availability that may be advantageous during the initial phases of wound healing, while the liposome-encapsulated fraction is released more gradually, supporting sustained tissue repair over time (Zhou et al., 2024). The mathematical modelling further demonstrates that these two release phases originate from distinct transport mechanisms: rapid concentration-driven diffusion of the unencapsulated fraction followed by controlled Fickian diffusion of the encapsulated compound through the phospholipid bilayer. Such effect could not be achieved with the extract solution alone, as VRB is highly hydrophilic and would otherwise rapidly diffuse in the wound exudate environment.

### 3.4 Cellular studies

To evaluate the suitability of the developed system for potential therapeutic application, the effect of the samples on cell viability was assessed on human cultured keratinocyte cells (HaCaT). Between the two liposomal formulations, BD-Extr-Lipo-A was selected for the *in vitro* cellular experiments, as it exhibited, although not statistically significant, a consistent trend toward higher encapsulation efficiency (Table 1) and more favorable colloidal stability compared to BD-Extr-Lipo-B (Figure 1).

**FIGURE 4:**
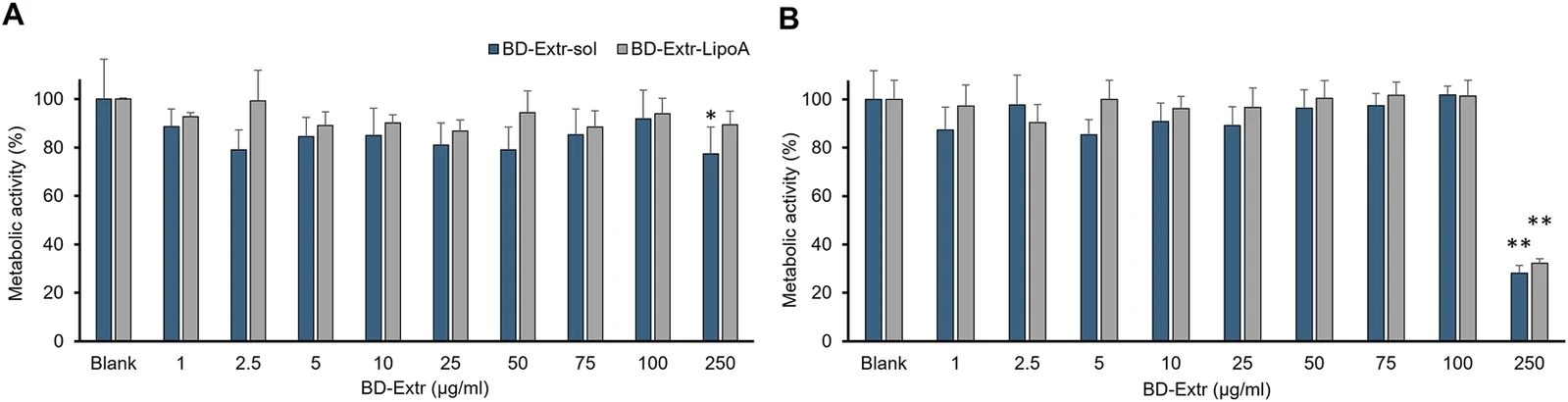
Biocompatibility study of Extract (blue bars) and VRB Lipo A (grey bars), determined by MTT essay after an exposure of 4 h **(A)** and 24 h **(B)** in complete medium. Data is reported as average ±SD, n = 5. Asterisks indicate statistically significant difference in metabolic activity of treated cells and blank (untreated cells) (*p < 0.05, **p < 0.01, One-way ANOVA with *post hoc* Tukey Test).

The biocompatibility of BD-Extr-sol and BD-Extr-LipoA was evaluated over a wide range of extract concentrations (1–250 μg/mL) after 4 h of treatment. As shown in Figure 4A, both the free extract and the liposomal formulation maintained high cell viability across most concentrations. This result is very promising regarding the potential applicability of this formulation in a wounded-skin environment, highlighting that the BD-Extr-LipoA formulation does not interfere with cell proliferation. A minor cytotoxic effect can be observed only in the BD-Extr-sol group at the highest concentration tested (250 μg/mL), where cell viability is slightly reduced to 77% (p < 0.05).

The biocompatibility of both BD-Extr-sol and BD-Extr-LipoA was also tested after 24 h of incubation, as shown in Figure 4B. In this case, the 250 μg/mL concentration showed significant cytotoxicity (p < 0.01), with the percentage of viable cells dropping to around 30%. This finding is consistent with other studies evaluating the cytotoxicity of VRB, the most abundant component in our extract. While short incubation periods with VRB enhance its scavenging activity and promote ROS elimination, also increasing the maximal oxygen consumption rate of mitochondria in stressed cells (Sciandra et al., 2023), longer incubation times can instead drive cells into a sub-apoptotic (sub-G1) state, interfering with the normal cell cycle and metabolism. This results in reduced mitochondrial activity and a lower signal in the MTT assay (Vasincu et al., 2020).

After confirming biocompatibility, the antioxidant activity was investigated at the cellular level to assess whether the formulations could reduce the excessive ROS generation associated with the wound microenvironment, where uncontrolled oxidative stress contributes to impaired healing and tissue damage (Lopes et al., 2024). To this aim, the DCFH-DA assay was performed. The DCFH-DA assay is a well-established method for quantifying intracellular ROS, as the probe can permeate the cell membrane and is oxidized by intracellular ROS to form the fluorescent derivative 2′,7′-dichlorofluorescein, which can be readily quantified (Zheng et al., 2025). HaCaT cells were pretreated with samples at two different extract concentrations (100 μg/mL and 250 μg/mL) for 4 h and then exposed to oxidative stress induced by hydrogen peroxide.

**FIGURE 5:**
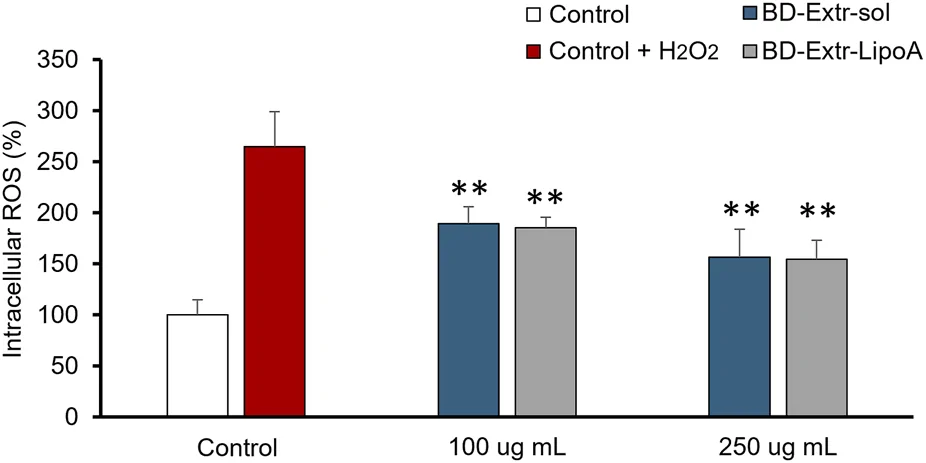
Assessment of intracellular ROS (%), after exposure to hydrogen peroxide and treatment with BD-Extr-sol (blue bars) and BD-Extr-LipoA (grey bars) at different concentrations of extract (100 and 250 ug/mL) for 4 h. White bar represents untreated cells, not exposed to oxidative stress. Red bar represents untreated cells, exposed to oxidative stress. Data is reported as average ± SD, n = 5. Asterisks indicate statistically significant difference with % of ROS in stressed cells treated by BD-Extr-Sol or BD-Extr-LipoA and control cells subjected to oxidative stress (red bar) (**p < 0.01, One-way ANOVA with *post hoc* Tukey Test).

As shown in Figure 5, the percentage of intracellular ROS was significantly reduced in cells pretreated with BD-Extr-sol and BD-Extr-LipoA compared with untreated cells exposed to the same oxidative environment (red bar, p < 0.01). Although ROS levels did not return to baseline values (grey bar) both treatments demonstrated a significant protective effect against oxidative stress. The comparable results achieved by BD-Extr-sol and BD-Extr-LipoA align with the moderate encapsulation efficiency within liposomes. In fact, the majority of VRB in liposomal formulation is readily available to drive its biological activity, just like in the case of BD-Extr-sol, while it could be speculated that the encapsulated fraction (maintenance dose, MD) could provide a sustained effect due to the controlled release from vesicles.

These findings − in line with the DPPH assay results − highlight that the liposomal formulation exhibits comparable antioxidant activity to the extract solution, indicating that encapsulation within lipid vesicles does not affect the scavenging capacity of the extract components.

### 3.5 Antimicrobial activity

**FIGURE 6:**
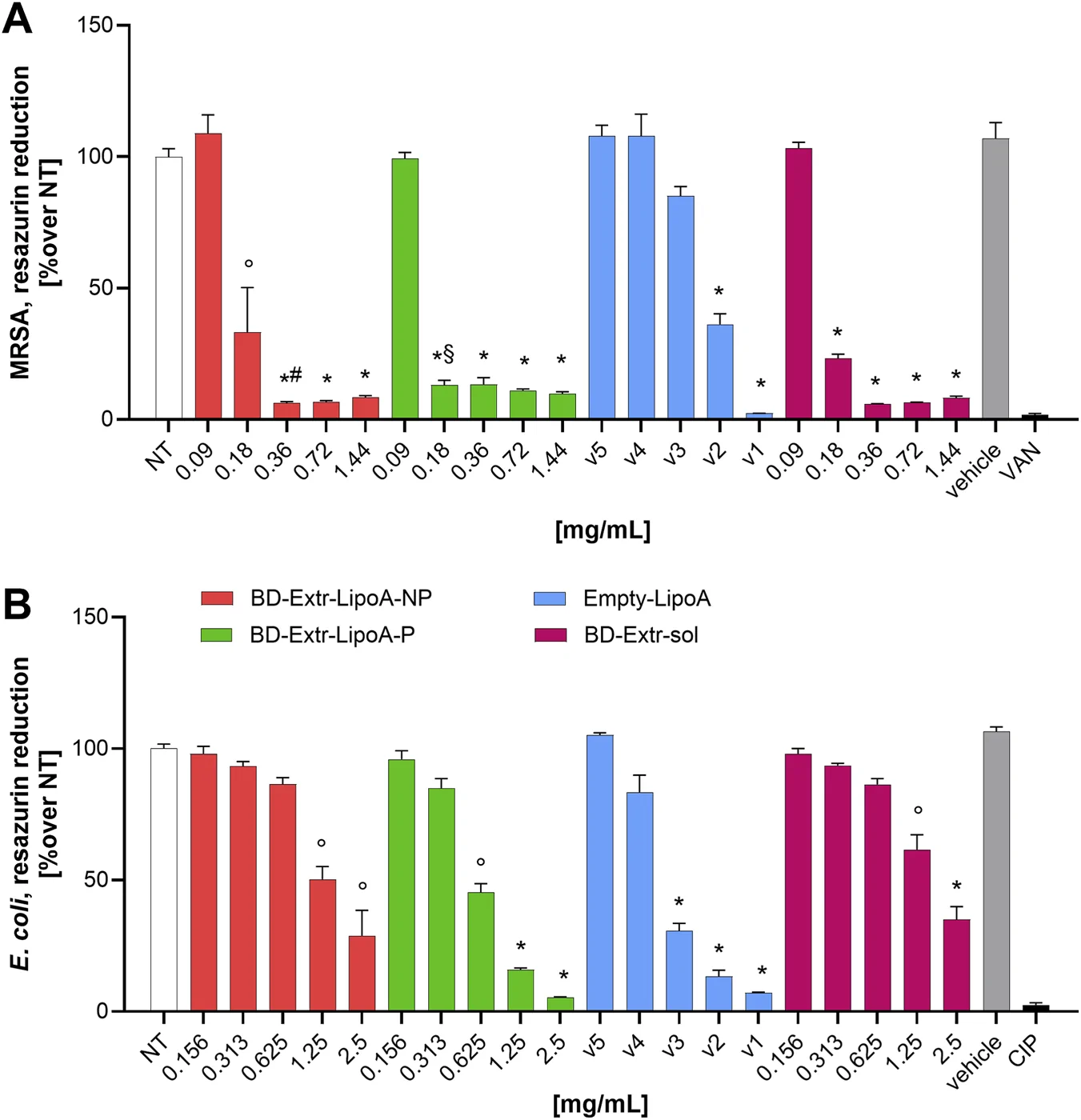
**(A)** MRSA and **(B)** *E. coli* viability after treatments at increasing concentrations of VRB: extract-loaded liposomal formulations unpurified (BD-Extr-LipoA-NP) and purified (BD-Extr-LipoA-P), empty liposomes (Empty-LipoA) or BD-Extr-sol. VAN: vancomycin at 4 μg/mL; CIP: ciprofloxacin at 1 μg/mL. Antibiotic concentrations were previously set in our laboratory. Fluorescence signals relative to resazurin reduction are expressed as percentages relative to non-treated (NT) controls. Empty-LipoA was used as controls at volumes (v1-v5) corresponding to the extract-loaded formulations. Data are presented as mean ± standard deviation (n = 3, in triplicate). ° denotes *P* < 0.05; *P < 0.02 vs. NT; ^§^ *P* < 0.02 vs. BD-Extr-sol, 0.18; ^#^ *P* < 0.02 vs. BD-Extr-LipoA-P, 0.36.

The resazurin reduction assay was used to evaluate the antimicrobial activity of the tested liposomal formulations and to determine the minimum inhibitory concentrations (MICs) against MRSA and *E. coli*. Fluorescence signals were expressed as percentages of the average signals in non-treated (NT) bacterial cultures. In MRSA cultures (Figure 6A), BD-Extr-sol reported a strong and, to some extent, comparable inhibitory effect to vancomycin (VAC) at VRB concentrations ranging from 1.44 to 0.18 mg/mL; 0.18 mg/mL was therefore identified as the MIC. The vehicle (CB) did not exhibit antimicrobial activity at any tested volume; therefore, only the highest volume used was reported in Figure 6. At the MIC, the extract loaded liposomal formulations showed a marked reduction in fluorescence signal, indicating that the extract retains its antimicrobial effects within the liposomes. Importantly, purified liposomes (BD-Extr-Lipo A-P) significantly reduced bacterial metabolic activity as compared to the BD-Extr-sol and the unpurified formulation (*P* < 0.05). Notably, Empty-LipoA exhibited antimicrobial effects at the higher volumes tested (volumes 1 and 2) but lost efficacy at the volume corresponding to VRB 0.18 mg/mL (v4).

In *E. coli* cultures (Figure 6B), BD-Extr-sol and BD-Extr-LipoA-NP showed a significant reduction in fluorescence at VRB concentrations of 2.5 and 1.25 mg/mL, even if only the highest concentrations reached the effects of ciprofloxacin (CIP). Interestingly, both the Empty-LipoA and BD-Extr-LipoA-P showed an antimicrobial effect even at lower concentrations (0.625 mg/mL), suggesting that the antibacterial activity observed against *E. coli* was not exclusively attributable to the encapsulated extract. In fact, as also reported by Zhang et al. (2017), phospholipids constituting lecithin-based vesicles may exert a synergistic antimicrobial effect against *E. coli*, enhancing bacterial inactivation independently of the active compound itself. This effect becomes more evident in purified vesicles, where the removal of the non-encapsulated extract allows the contribution of the phospholipids to be more clearly distinguished, whereas in unpurified formulations the strong effect of the free extract immediately available tends to mask this phospholipid-mediated antibacterial activity.

**FIGURE 7:**
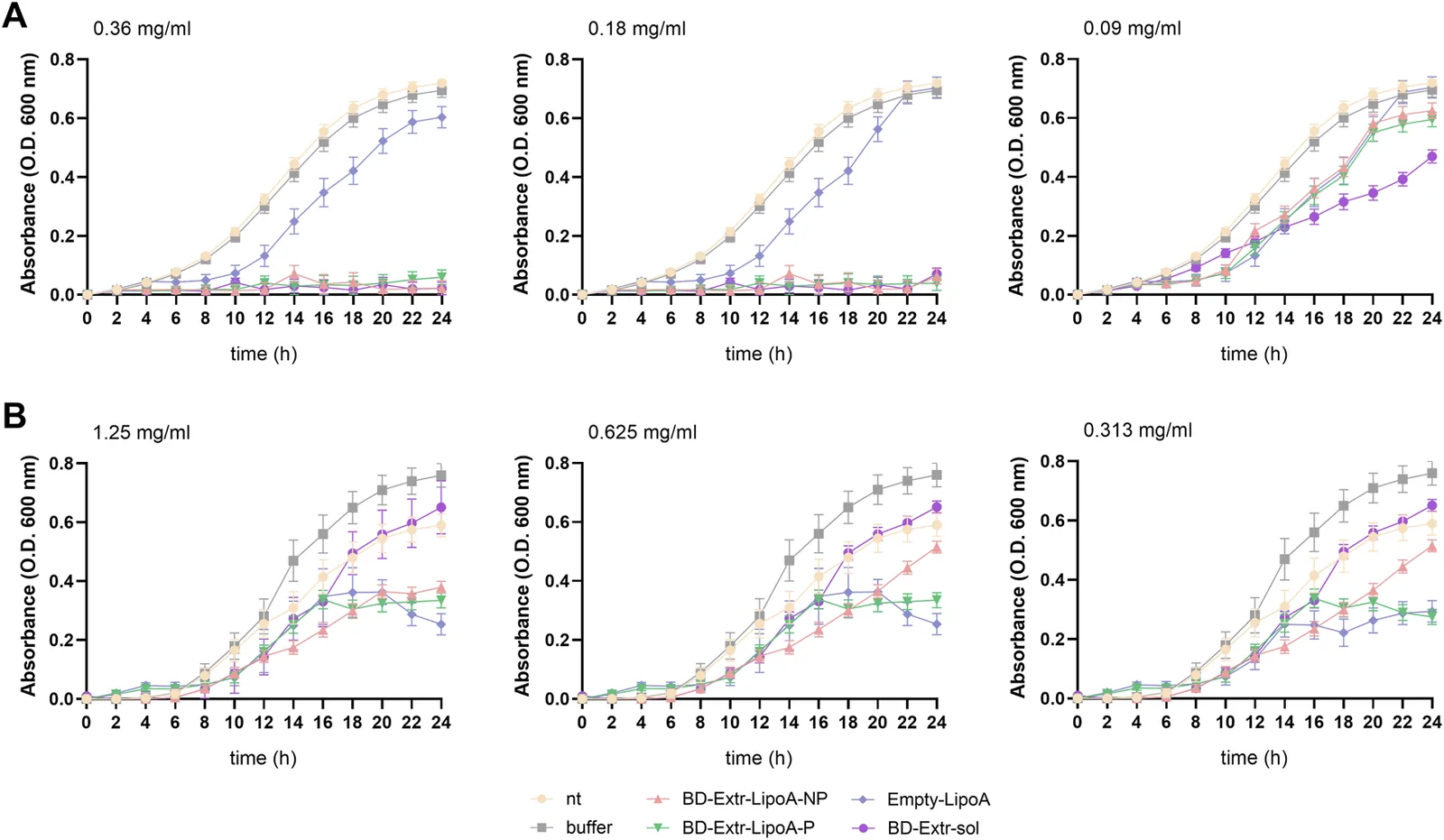
Kinetic growth curves of **(A)** MRSA and **(B)** *E. coli* cultured in the presence of the extract solution (BD-Extr-sol), extract-loaded liposomal formulations (BD-Extr-LipoA-NP, BD-Extr-LipoA-P), empty liposomes (Empty-LipoA), or vehicle (buffer). Bacterial growth was monitored over 24 h at 37 °C by measuring optical density (OD_600_). Each panel represents a different VRB concentration. The non-treated control (NT) served as the negative control. Images are representative of three independent experiments.

To track the bacterial growth over time, kinetic growth curves were recorded over 24 h at 37 °C. In MRSA cultures (Figure 7A), the kinetic growth curves of bacteria incubated with Empty-LipoA or vehicle closely overlapped with the NT curve, indicating the absence of antimicrobial activity. In contrast, BD-Extr-LipoA-NP, BD-Extr-LipoA-P and BD-Extr-sol at 0.36 and 0.18 mg/mL VRB completely inhibited bacterial growth over 24 h of incubation, demonstrating long-lasting and consistent bactericidal effects. At 0.09 mg/mL, VRB in liposomal formulations exhibited a delay (approximately 4 h) in the lag phase of MRSA growth, which, along with the data reported in Figure 6, is indicative of bacteriostatic activity at this specific concentration.

In *E. coli* cultures (Figure 7B), the growth curves indicate primarily bacteriostatic activity for both the extract solution and the extract-loaded liposomes. Indeed, bacterial growth was observed for all samples, and liposomes at 1.25 mg/mL delayed the lag phase. As reported in Figure 6B, the effects of Empty-LipoA are indistinguishable from the VRB liposomal formulation, confirming the significant contribution of the phospholipids to the antibacterial response in *E. coli*.

To further validate the antimicrobial activity observed in the viability assays, colony-forming unit (CFU) counts were performed (data not shown). In MRSA cultures, CFU enumeration confirmed the results obtained with the resazurin assay, showing a marked reduction in bacterial colony counts at 0.18 mg/mL of VRB administered as BD-Extr-sol or BD-loaded liposomal formulations, indicating that encapsulation did not impair antimicrobial efficacy. CFU counts in *E. coli* cultures showed no significant differences between empty liposomes and VRB-loaded liposomes, with similar colony numbers across treatments. These findings are consistent with the resazurin assay and growth curve data, confirming the limited antimicrobial effect of VRB and its formulations against *E. coli* under the tested conditions.

The reduced antimicrobial activity observed against *E. coli* compared with methicillin-resistant *S. aureus* is primarily attributable to fundamental differences in bacterial cell envelope architecture. As a Gram-negative organism, *E. coli* possesses an outer membrane enriched in lipopolysaccharides (LPS), which constitutes a well-known permeability barrier that can limit the penetration of both the free extract and the nanocarrier-based systems (Sang et al., 2022). This structural feature is widely recognized as a key determinant of the intrinsic resistance of Gram-negative bacteria to many antimicrobial agents, including lipid-based delivery systems. In contrast, the absence of an outer membrane in Gram-positive MRSA enables more direct interaction between liposomal carriers and the cytoplasmic membrane, thereby facilitating BD delivery and enhancing antimicrobial efficacy (Rizkita et al., 2024). In addition to structural considerations, the physicochemical properties of liposomal formulations, such as surface charge, may further influence their interactions with bacterial cells. The outer membrane of Gram-negative bacteria is highly negatively charged due to LPS, which can result in electrostatic repulsion when liposomes exhibit a similar zeta potential. This effect may limit adhesion and fusion of liposomes with the bacterial surface, thereby reducing intracellular delivery of VRB. Conversely, Gram-positive bacteria lack the additional outer membrane barrier, allowing more efficient interaction with lipid-based carriers.

In our experiments, *E. coli* cultures were incubated with liposomal formulations at concentrations higher than those used in MRSA cultures. Increased liposome concentrations may directly reduce VRB variability, potentially due to aggregation or partial destabilization. These factors alone are unlikely to explain the reduced antimicrobial activity observed in *E. coli*. Indeed, our results are consistent with previous reports indicating that Gram-negative bacteria exhibit lower susceptibility to liposomal antimicrobial systems, primarily due to the protective role of the outer membrane (Cheung Lam et al., 2016).

Although the present study demonstrates the antibacterial activity of the verbascoside liposomal formulation, the underlying mechanism was not investigated in detail. Previous studies have suggested that verbascoside exerts antimicrobial effects through multiple mechanisms, including disruption of bacterial membrane integrity, increased membrane permeability, induction of oxidative stress, and interference with essential cellular processes (Marčetić et al., 2025). In addition, liposomal encapsulation may enhance antibacterial efficacy by improving the stability of verbascoside, promoting its interaction with the bacterial surface, and facilitating the local delivery of the bioactive compound.

## 4 Conclusion

In conclusion, this study demonstrates that liposomal encapsulation is an effective strategy to prolong the release of hydrophilic plant extracts, with potential benefits in the context of wound healing. The developed formulations successfully incorporated the verbascoside-rich *Buddleja davidii* extract, while preserving its chemical stability, antioxidant properties, and biocompatibility toward human keratinocytes. Most importantly, the liposomal system enabled a controlled and sustained release of verbascoside, addressing a key limitation of the free extract, which − owing to its highly hydrophilic nature − would otherwise rapidly diffuse within the wound exudate and be readily cleared from the application site. The observed dual release profile, consisting of an initial readily available fraction followed by a prolonged release from the vesicles, may be particularly beneficial in modulating both early oxidative stress and later stages of tissue repair; a mechanism that remains to be validated in preclinical experiments. In addition, the formulations displayed a marked strain-dependent antimicrobial profile *in vitro*, characterized by higher activity against MRSA, while *E. coli* cultures showed less susceptibility and a predominantly bacteriostatic response. From a translational perspective, future studies will be required to evaluate the *in vivo* efficacy of the formulation in promoting tissue regeneration and modulating inflammation using appropriate animal models.

## Data Availability

The raw data supporting the conclusions of this article will be made available by the authors, without undue reservation.
