# Supplementary material for: Verbascoside-rich Buddleja davidii extract in liposomal systems: modulation of release behavior for topical wound treatment
Source: Front Bioeng Biotechnol. 2026 Aug 26;14:1903742. doi: 10.3389/fbioe.2026.1903742 (PMC13553915; doi:10.3389/fbioe.2026.1903742)
Supplement: Supplementary file 1 [file Supplementaryfile1.docx]

Supplementary Material

**Supplementary Figure 1.** Chemical stability of BD-Extract and liposomes (BD-Extr-LipoA and BD-Extr-LipoB) over 60 days. The concentration of verbascoside (ng/µL) was measured at day 0, 30, and 60 through HPLC. Samples were stored at 4 °C. Results are stated as mean ± standard deviation (n = 3).

**Supplementary Table 1.** In vitro release kinetic parameters fitted to different mathematical models

|  |  | **Formulation** | | | | |
| --- | --- | --- | --- | --- | --- | --- |
|  |  |  | **Unpurified** | | **Purified** | |
| **Kinetic model** |  | **Extract** | **VRB Lipo A** | **VRB Lipo B** | **VRB Lipo A** | **VRB Lipo B** |
| **Zero-order** | **R²** | 0.443 | 0.471 | 0.457 | 0.812 | 0.819 |
|  | **k₀** | 9.77 | 9.24 | 9.17 | 3.94 | 2.87 |
| **Pseudo-first-order** | **R²** | 0.991 | 0.985 | 0.979 | 0.865 | 0.849 |
|  | **k₁** | 1.34 | 0.774 | 0.882 | 0.052 | 0.031 |
| **Pseudo-second-order** | **R²** | 0.975 | 0.958 | 0.942 | 0,833 | 0.810 |
|  | **k_p_₂** | 0.020 | 0.013 | 0.011 | 0.001 | 0.001 |
| **Higuchi** | **R²** | 0.690 | 0.718 | 0.706 | 0.994 | 0.996 |
|  | **k_H_** | 36.410 | 35,847 | 36.826 | 11.552 | 8.363 |
| **Korsmeyer–Peppas** | **R²** | 0.815 | 0.831 | 0,819 | 0.997 | 0.998 |
|  | **k_KP_** | 0.890 | 0.722 | 0.781 | 0.131 | 0.114 |
| **Hixson–Crowell** | **R²** | 0.822 | 0.835 | 0.819 | 0.848 | 0.839 |
|  | **k_hc_** | 0.5123 | 0.311 | 0.374 | 0 021 | 0.013 |
| **Weibull** | **R²** | 0.994 | 0.995 | 0.991 | 0.988 | 0.985 |
|  | **β (shape parameter)** | 0.32 | 0.44 | 0.39 | 0.54 | 0.56 |
|  | **α (scale parameter)** | 0.26 | 0.32 | 0.29 | 31.43 | 31.43 |
